# Hypermetabolic lymphadenopathy on positron emission tomography scan following COVID‐19 vaccination: A mimicker of disease progression in Hodgkin lymphoma

**DOI:** 10.1002/jha2.268

**Published:** 2021-07-22

**Authors:** Adam Suleman, Alexander Bilbily, Matthew Cheung, Lisa Chodirker

**Affiliations:** ^1^ Department of Medicine University of Toronto Toronto Canada; ^2^ Department of Medical Imaging Sunnybrook Health Sciences Centre Toronto Canada; ^3^ Division of Hematology Department of Medicine Sunnybrook Health Sciences Centre Toronto Canada

## Description

A 38‐year‐old woman with advanced stage classical Hodgkin lymphoma presented for her interim ^18^F‐ fluorodeoxyglucose (FDG) positron emission tomography (PET) scan after completing two cycles of doxorubicin, bleomycin, vinblastine and dacarbazine (ABVD). Her interim PET after two cycles of ABVD showed response in the previously identified hypermetabolic lymphadenopathy (*PET on presentation shown in panel A*), but interval development of FDG avidity anterior to the right proximal humerus and axilla (*panel B*), prompting the consideration to dose‐intensify her therapy. However, the patient clarified that she had received the COVID‐19 vaccine (Pfizer) in her right arm seven days prior to the PET scan. Repeat PET two weeks later showed improvement in the right axillary hypermetabolic lymph nodes (*panel C*), favouring vaccine‐related reactive hypermetabolism. Recognizing the ability of vaccines to induce PET‐avid hypermetabolic lymphadenopathy is crucial to avoid misinterpreting vaccine‐related responses with interim PET positivity that would have altered treatment.

**FIGURE 1:**
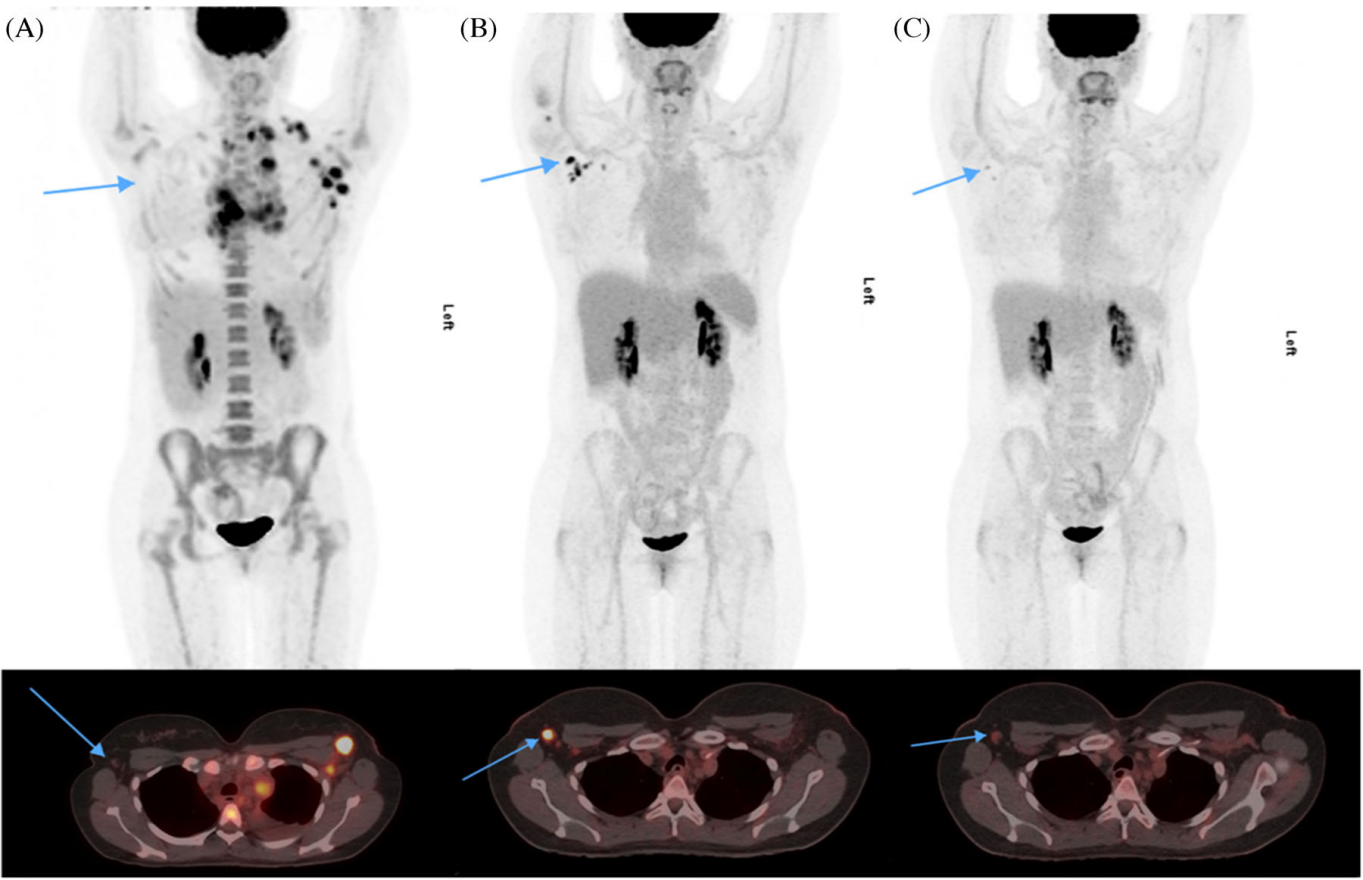
Pre‐treatment (panel A), interim (panel B) and repeat two‐weeks after vaccination (panel C) positron emission tomography scans

